# Differentiation of neurosphere after transplantation into the damaged spinal cord

**DOI:** 10.25122/jml-2022-0346

**Published:** 2023-05

**Authors:** Svetlana Mykolaiivna Gramatiuk, Yulia Viktorovna Ivanova, Arsen Arsenievich Hudyma, Karine Sargsyan, Igor Andreevich Kryvoruchko, Inna Sergeevna Puliaieva

**Affiliations:** 1. Department of Biotechnology, Institute of Bio-Stem Cell Rehabilitation of the Ukrainian Association of Biobanks, Kharkiv, Ukraine; 2. Department of Biotechnology, Louisiana State University, Baton Rouge, Louisiana, USA; 3. International Biobanking and Education, Medical University of Graz, Graz, Austria; 4. Department of Surgery No.1, Kharkiv National Medical University, Kharkiv, Ukraine; 5. Emergency Medical Care, Ternopil National Medical University named after I. Ya. Gorbachevsky, Ternopil, Ukraine; 6. Department of Medical Genetics, Yerevan State Medical University, Yerevan, Armenia; 7. Department of Surgery No.2, Kharkiv National Medical University, Kharkiv, Ukraine

**Keywords:** neurospheres, stem cells, directed differentiation, spinal cord injury

## Abstract

This study aimed to compare the differentiation and survival of human neural stem/progenitor cells of various origins in vitro and after transplantation into the injured spinal cord of laboratory animals. Rats with simulated spinal cord injury were transplanted with neurosphere cells obtained by directed differentiation of HUES6 cell lines. Fluorescence microscopy was used to visualize the obtained results. HUES6#1 and iPSC#1 neurospheres showed a wide range of markers associated with glial differentiation. The expression of the proliferation marker Ki67 did not exceed 25%, both in the lines of early and late neurospheres. Although neurospheres did not fully differentiate into astrocytes in vitro, they massively approached the GFAP+ astrocyte phenotype when exposed to the transplanted environment. PSC-derived neurospheres transplanted into the site of SM injury without additional growth factors showed only moderate survival, a significant degree of differentiation into astrocytes, and moderate differentiation into neurons. The difference in the survival and differentiation of HUES6#1 and iPSC#1 neurospheres, both in vitro and in vivo, can be explained by the difference in the regulatory behavior of signaling molecules corresponding to the source of origin of PSCs. Derivatives of human PSCs of various origins obtained according to the described differentiation protocol did not mature into astrocytic populations, nor did the glycogenic transition of PSC-derived NSCs occur in vitro. The study demonstrated the impact of the injured spinal cord microenvironment on the differentiation of transplanted HUES6#1 and iPSC#1 into astrocytes. The results showed that HUES6-derived neurospheres generated 90% of GFAP+ astrocytes and 5-10% of early neurons, while iPSC-derived neurospheres generated an average of 74% GFAP+ astrocytes and 5% of early neurons in vivo.

## INTRODUCTION

Motor deficits and disability often accompany spinal trauma involving annotation methods or prosthetics. The field of medicine related to neuron transplantation of stem cells (SC), analysis of their survival and behavior after transplantation, and the study of the influence of SC on compensatory processes in experimental models of brain damage in laboratory animals are rapidly developing [1-5].

Spinal cord injury (SCI) is characterized by damage to axons, degeneration of the nervous system, formation of cystic cavities, and increased activity of neurogenesis-inhibiting and pro-inflammatory factors [5,6]. To protect the tissue surrounding the injury site, fibroblasts and reactive astrocytes form an impenetrable barrier that prevents the endogenous regeneration of neurons and glial cells in the affected area [7, 8]. Numerous studies demonstrate the long-distance growth of axons derived from transplanted neural stem cells (NSCs) after TCM. Therefore, neural stem cells derived from various sources are considered a promising source of cells for reconstructing the injured spinal cord (SCI) due to their potential for neuroprotection, neuroregeneration, remyelination, and replacement of lost nerve cells, which can restore the connection of damaged axon.

It has been shown that pure populations of microglia cells [8], astrocytes [9, 10], and oligodendrocytes [11] can be obtained from induced pluripotent stem cells (iPSC), which is an acceptable method of getting a large number of neural progenitors - astrocytes, neuroblasts, neurons, etc., in vitro for autologous transplantation. The generating neurons of different types are also essential for modeling neurodegenerative diseases, TSM, and developing methods to combat their consequences. Transplantation of neurons to the damaged CM and their differentiation in the damaged area allows damaged neurons to connect with their targets, so they cannot survive. The function is successful in the damaged environment without supporting an astrocyte substrate, but today, many works have been devoted to the problem of generating astrocytes from human PSC [9,12-15]. The results of Krencik et al. (2011) [9, 13] proposed a method for creating large quantities of pure astrocyte populations from PSCs based on the principle of "gliogenic transition" of developing NSCs.

The reprogramming of somatic cells into a pluripotent state was initially achieved through the overexpression of transcription factors, including Oct4, Sox2, Klf4, NANOG, and Myc, using integrating viral vectors [16]. However, current protocols for somatic cell reprogramming involve integration-free methods, such as the delivery of Yamanaka factors [17,18], to avoid host cell genome mutations and prevent the oncogenicity of transplanted PSCs.

It is important to carry out NSC transplantation in the injured CM within a specific time frame. Previous studies suggest that the optimal time window for transplantation is within four days of the subacute phase of CM [2,19]. Transplanted NSCs can create a microenvironment necessary for the regeneration of recipient axons, which gives hope for restoring connection after TCM. Creating an environment for regenerating damaged axons requires a substrate with a neuronal identity that can perform physical and metabolic support to developing neurons and support the homeostasis of the nervous tissue as a whole, including its network activity. Recent studies on the transplantation of both NSCs and differentiated SCs of various origins have shown that the cells induce plasticity. The regeneration of the injured spinal cord promotes the reconstruction of neural circuits by forming synapses between host neurons and graft-derived neurons, secreting neurotrophic factors to promote axonal elongation, and reducing retrograde axonal degeneration [19-21].

One of the distinctive properties of SCs, in addition to self-maintenance and multipotency, is their ability to form neurospheres under the conditions of cultivation with mitogens. Neurospheres are unique cellular microsystems with specific spatial and functional cellular organization and intercellular interactions, where biochemical signals are necessary for survival and proliferation. The differentiation of progenitors within neurospheres can give rise to three types of cells found in the nervous system: neurons, astrocytes, and oligodendrocytes [9].

Neurospheres formed from human neural stem cells in tissue culture have different cellular compositions and architecture. In some, the cells are arranged chaotically, while in others, structures in the form of rosettes are found, which have a central cavity with cells radially diverging from it and arise in monolayer cultures of neuronal progenitor cells obtained from human pluripotent stem cells.

Rosettes persist for a long time in culture and after transplantation of cultures into the brain. Rosette cells are capable of symmetrical and asymmetrical division, express nestin (a marker of stem cells), have a villous apparatus, and are connected in the central part by desmosome-like contacts. Rosette formation imitates the behavior of cells at the beginning of the neural tube [22,23]. Processes of non-expressing cells in rosettes are oriented radially. Cells that migrate beyond the rosette express β-Tubulin III, a marker of early neuroblasts. The specific neuroepithelial organization of the sockets is unique and necessary for the long-term support of NSCs in culture conditions.

Neurospheres are a convenient model not only for studying the mechanisms of neural differentiation and gene expression but also for the influence of microenvironmental signals that regulate the phenotypic specialization of neural tissue cells in vitro and in vivo [24]. During the development of NSCs in tissue culture, specific patterns of cell behavior at different stages of differentiation were revealed, and it was established that the transition from the stem state to the differentiated state occurs for neurons and glia in different ways [9]. Therefore, for the prospects of further practical application, it is necessary to consider the results of transplantation studies on the use of NSCs in models of brain damage.

Thus, the study aimed to compare the differentiation and proliferation in cultures of human neural stem/progenitor cells of various origins in vitro and in vivo after transplantation into the injured spinal cord of laboratory animals.

## MATERIAL AND METHODS

The study was conducted in compliance with the ethical guidelines outlined in "On the Protection of Animals from Cruel Treatment" (No. 3447-IV dated 21.02.2006) and the "European Convention for the Protection of Vertebrate Animals Used for Experimental and Other Scientific Purposes" (86/609 EEC, Strasbourg). The Bioethics Committee of the Ukrainian Association of Biobanks reviewed and approved the research protocol (protocol No. 2003/2021, dated 11.11.2021).

Lines HUES6 (line 6 of embryonic stem cells (ESC)) and induced pluripotent stem cells Human Induced Pluripotent Stem Cells (iPSC) were obtained under the agreement of scientific and practical cooperation (Project number 4009_17 Biobank Graz, Austria).

In this study, male Wistar rats aged 3 months and weighing 160±12 g were used. All animals were modeled with USM and transplanted PSCs, differentiated according to a standard protocol. The animals were divided into four groups for transplantation: Group I - HUES6 with the addition of growth factors (Complete, Roche Diagnostics GmbH, Germany) (n=6); Group II - HUES6 with the addition of PBS/1% glucose (without the addition of growth factors) (n=6); Group III - iPSCs with the addition of growth factors (n=6); Group IV - iPSCs with the addition of PBS/1% glucose (without the addition of growth factors) (n=6).

### Cultivation of iPSCs

PSCs were cultured in 6-well plates (Nunc® Matrigel™, BD Biosciences, Thermo Scientific, USA) in a CO2 incubator under conditions of humidified air with 5% CO2 at a temperature of +370 C in the TeSR-E8 environment (StemcellTM Technologies, Thermo Scientific, USA). A solution of trypsin and EDTA (Hyclone, New Zealand) was used for transplanting PSC cultures. The nutrient mediFibro Stage system (USA) replaced the nutrient medium automatically neurospheres. Neuroblast cells were obtained after PSC cultures reached 80% confluency of the monolayer: homogeneous elements of medium-sized colonies were transferred to 6-well plates of ultra-low adhesion (Stemcell TM Technologies, USA) at a concentration of 104 cells/ml and cultivated in TeSR-E8 medium. The culture medium was replaced with TeSR-E8 medium and DMEM F-12 Gibco (Thermo Fisher Scientific, USA)/Ham's F-12 (Thermo Fisher Scientific, USA) in a ratio of 1:1 with 40 times the content of growth factors (Complete, Roche Diagnostics GmbH, Germany), 1% Gibco N-2 Supplement (Thermo Fisher Scientific, USA) and 1% Gibco B-27 Plus Neuronal Culture System (Thermo Fisher Scientific, USA).

**Figure 1.**
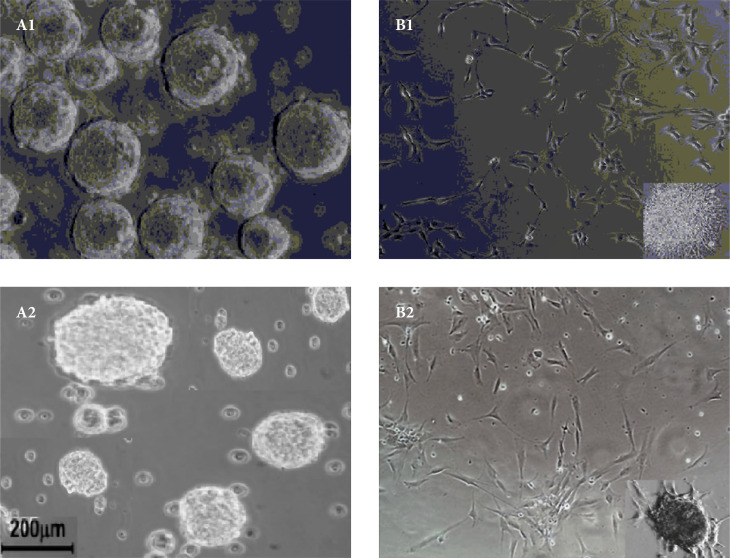
Photomicrographs of derivatives of PSK neurospheres (A1, B1 - HUES6#1; A2, B2 - iPSK#1) according to phase-contrast microscopy data: A - after 7-10 days of cultivation; B - after 10-12 days of cultivation (scale lines)

Cells in the culture medium were distributed 2 ml into each well of a six-well plate with an anti-adhesive coating. The final cell density was an average of 1000 cells/cm2. Every 2-3 days, 50 μl of 40-fold growth factors were added. Neurospheres were visualized using a phase-contrast microscope on the 7-10th day. Real neurospheres were characterized by an almost perfect spherical shape and precise, phase-bright outer edges (Figure 1 A1, A2). After 10-12 days of cultivation (3-4 passages), rosette-like structures appeared in the culture (Figure 1 B1, B2).

A confluent culture of neuroblast cells, enriched in stem and progenitor, was represented by a heterogeneous group of large cells expressing stemness markers, which were determined using the Bio-techne FMC-020 kit (UK) according to the instructions and the minimum requirements for the determination of stemness identity cells [25,26]. Additionally, markers of mesenchymal and neuroblast differentiation, such as Oct4, Vimentin, CD34, CD117, Desmin, E-cadherin, and CKW were also present in the culture. The cells had high proliferative activity according to Ki 67 according to the international standard ISO 20387: 2018

### Neuronal induction of human PSC

The standard directional differentiation protocol proposed by R. Krencik et al. was used in this study (2011) [13] to obtain iPSK#1 and HUES6#1. The NSCs were pre-cultured for two weeks to primary neurospheres using fetal bovine serum (FBS) and growth factors (EGF/FGF2), which were definitively differentiated into secondary neurospheres. For the following two weeks, FBS and astrocyte maturation inducers CNTF, BMP2/4, and FGF1 were used.

### Immunohistochemical studies

Immunohistochemical studies of neurospheres were performed after their fixation in a 4% paraformaldehyde solution. Fixed cells were permeabilized with a 99% alcohol solution followed by blocking with a 5% bovine serum solution (Sigma, USA) and incubated with primary antibodies for 12 h. at a temperature of +4 °C. Then the cells were washed with a resolution of secondary antibodies for 40 min. Immunophenotyping of the obtained cultures was performed in the 3rd passage. The presence of specific marker proteins was determined in the cultures: neurons – β-Tubulin III; NSC – Nestin; neural PCs – Vimentin; and glial cells – GFAP. Mouse monoclonal anti-β-Tubulin III antibodies (Sigma, USA), mouse monoclonal anti-GFAP antibodies (Abcam, UK), mouse monoclonal anti-Nestin antibodies (Abcam, UK), monoclonal anti-Vimentin antibodies were used as primary antibodies. Secondary antibodies were immunoglobulin G conjugated with CNTF, BMP2/4, and FGF1 activators (Sigma, USA) from standard kits according to the instructions. Nuclei of nonviable cells were stained with DAPI (4′,6-diamidino-2-phenylindole) (Thermo Scientific, USA). An EVOS FL fluorescent microscope (Life Technologies, USA) was used to visualize cell cultures and histological preparations.

### Polymerase Chain Reaction

The reverse transcription polymerase chain reaction (RT-PCR) of neurosphere cell markers was performed using equipment manufactured by Bio-Rad Laboratories, Inc. (USA). RNA extraction was performed using the Bio-RadRNA Kit (Bio-Rad Laboratories, Inc., USA) according to the manufacturer's protocol.

### Spinal cord injury

Spinal cord injury (SCI) in rats was simulated using an incomplete transverse section of the spinal cord [27]. Rats were anesthetized by inhalation of 5% halothane, which was reduced to 2% during surgery, in combination with a mixture of nitrous oxide/oxygen (1:2, v/v). A dorsal laminectomy was performed to identify the segment of the spinal cord at the L3-5 level of the spine. The spinal cord was cut laterally using microsurgical scissors. After the operation, the muscles and skin were sutured in layers, and an antibiotic (gentamicin, 1000 μl) was injected subcutaneously. Reproduction of the model was considered adequate if paralysis of one or both upper limbs was observed a day after the operation.

One week after the injury, PSC-derived neurospheres were transplanted into the injury site. Animals were analyzed 17 and 27 days after transplantation to examine the relative distribution and regenerative properties of the cells in the USM site.

### Transplantation of neurospheres

The viability of neurospheres, which was 90±5%, was determined before their introduction using DAPI staining. Cell counting was performed on a BioRad (USA) automatic analyzer. Neurospheres were transplanted into rats via LP spinal puncture at L3-5 spinal level under anesthesia. One week after a spinal cord injury simulation surgery, 2×106 neurospheres were introduced in 40 μl of culture medium. To wash the contents of the needle, 10 μl of culture medium was slowly injected into the intrathecal space through a 25-gauge neonatal needle, after which the skin was sutured. Cyclosporin-A (15 mg/kg, Sandimmune, Novartis, Canada) was administered subcutaneously to the animals every day from the day of transplantation until withdrawal from the experiment.

### Statistical analysis

The obtained data were processed using the Statistica software package, version 10.0 (Stat Soft Inc., USA). The results of statistical data processing are presented as mean±standard error of the mean. To assess group differences, either the parametric Student's t-test or the non-parametric U-Mann-Whitney test was used. The results were considered statistically significant when the p-value was equal to or less than 0.05.

## RESULTS

Based on the data obtained from the study of the mechanism of neuronal development in vitro, we focused on the generation of neurospheres from human PSCs of various origins (ESCs and iPSCs). Cells were then transplanted (with or without the addition of growth factors) to the injured spinal cord of rats for comparative characteristics of their survival and differentiation. Additionally, we used inducers of astrocytic maturation (CNTF, BMP2/4, and FGF1) to generate a population of immature astrocytes from the human PSCs.

### Characterization and differentiation of PSC-derived neurospheres in vitro

We performed homogeneous immune labeling of human PSC neurospheres derived from iPSCs and HUES6 using astrocytic marker Vimentin, transcription factor HoxB4, and neuroblast markers Sox2 and Nestin.

**Figure 2.**
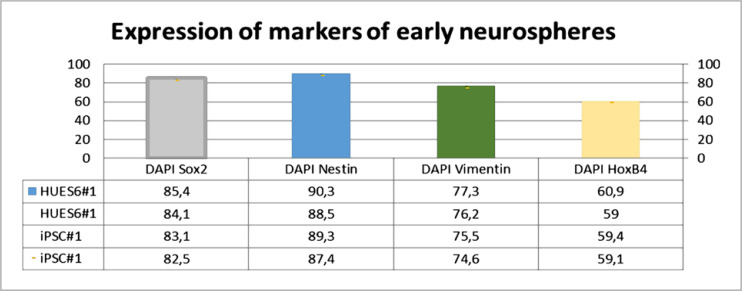
Expression of early neurosphere markers (DIV 60-80): - HUES6#1, - iPSK#1. (NSC markers Sox2 and Nestin, early astrocytic NSC marker Vimentin, and transcription factor HoxB4); n=6; p≤0.05

Figure 2 shows the expression of early neurospheres markers (DIV 60-80), including NSC markers Sox2 and Nestin, early astrocytic NSC marker Vimentin, and the transcription factor HoxB4, which is expressed in the developing spinal cord, indicating the successful stability of neuro-epithelial cells [9, 27].

Thus, in vitro, neurosphere generation results showed that HUES6#1 and iPSK#1 are examples of neuronal induction and stability.

When analyzing the culture of neuroblast cells at 3-4 (early) and 7-8 (later) passages, neurospheres were readily dissociated, and the cells were deposited on a Poly-L-Ornithine/Laminin coated cover glass (Corning® BioCoat® USA) and exposed to an inducer. CNTF was used to induce astrocytic maturation for one week, following the original protocol [9,13]. However, this approach proved to be insufficient to stimulate astrocyte maturation. At the end of a week, none of the differentiated neuroblast culture lines produced appreciable numbers of GFAP+-positive astrocytes, so differentiation was extended to two weeks. After two weeks of differentiation, neurocytes, as expected, turned into a homogeneous culture of astrocytes, although they homogeneously expressed the early astrocytic marker vimentin.

**Figure 3.**
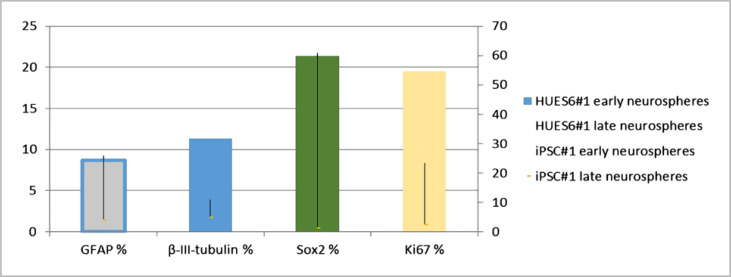
Expression of markers by differentiated neurospheres derived from HUES6, expressed as a percentage relative to DAPI+-nuclei, in medium with CNTF, after two weeks of cultivation: A - astrocyte marker GFAP, B - neuronal marker β-III-tubulin, C - NBK marker Sox2, D - proliferation marker Ki67; early neurospheres - DIV 75-95, late neurospheres - DIV 170-180. n=6; p≤0.05

Analysis of the Sox2 marker in the culture of neuroblasts revealed a significantly higher content in NSCs DIV~170-180, in contrast to NSCs DIV 75-95, and the culture of NSCs derived from HUES6, in comparison to NSCs derived from iPSCs (Figure 3 A, B). The obtained data suggest lower suitability of neurospheres for urgent (within a week) differentiation.

The iPSC-derived late progenitor cells did not differentiate into astrocytes or neurons and showed a low expression level of Sox2 and Ki67 markers (Figure 3 C, D), although the expression of the Vimentin marker remained unchanged. Expression of the proliferation marker Ki67 by lines of both early and late neurospheres did not exceed an average of 25% and tended to decrease progenitor cells derived from iPSCs (Figure 3 D).

In the differentiation process, neurospheres did not necessarily give rise to homogeneous monolayer cultures but were also partially represented by sections from densely packed colonies of neuroblastic Sox2+ cells.

In general, differentiated PSC derivatives from different sources in vitro showed the early astrocyte marker Vimentin, the astrocyte marker GFAP, the neuronal marker β-III-tubulin, and the NSC marker Sox2, and the proliferation marker Ki67. However, despite using our original differentiation protocol, we could not obtain pure cultures of astrocytes from any of the PSC lines.

### Transplantation of PSC-derived neurospheres to the injured spinal cord

Although the differentiation of neurospheres in our study did not lead to the formation of pure cultures of astrocytes in vitro, the survival and differentiation of transplanted cells were greatly influenced by the host environment's signaling agents, i.e., the TCM cell.

**Figure 4.**
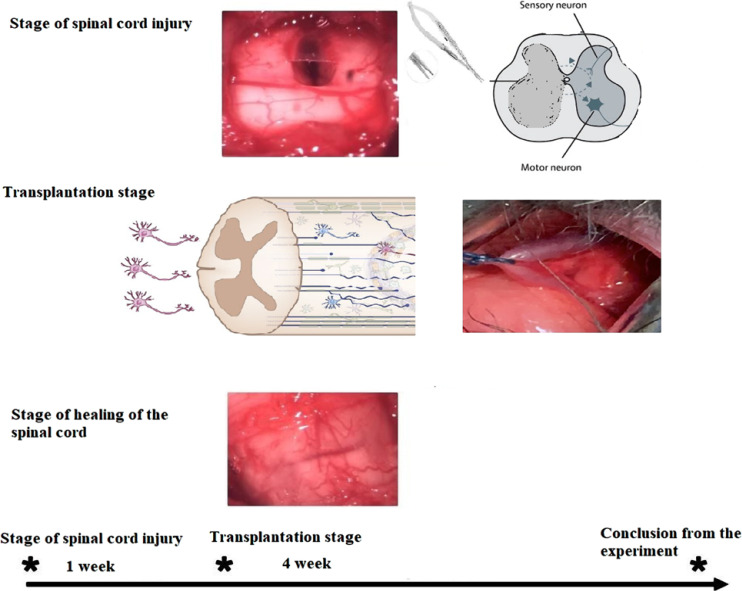
Scheme of SCI simulation and transplantation of neurospheres derived from PSCs from different sources to the damaged spinal cord of a rat

DIV~130 neurospheres, derived from HUES6 and iPSCs, were transplanted into rats one week after lateral SC injury at the L3-5 spinal level, as shown in Figure 4.

**Figure 5.**
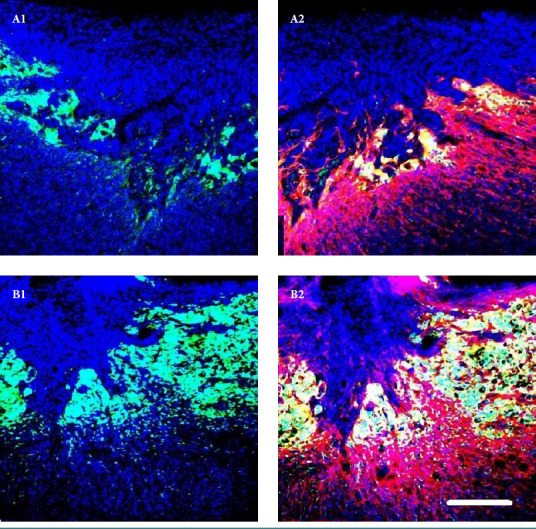
An example of the survival of HUES6#1 derived neurospheres in the injured spinal cord four weeks after SCI and transplantation: A – without additional growth factors (A1 – DAPI/hNUC; A2 – DAPI/hGFAP), B – with different growth factors (B1 - other DAPI/hNUC; B2 – DAPI/hGFAP). Scale bar: 500 μm

To assess the potential effects of growth factors on the survival and differentiation of HUES6- and iPSC-derived cells, neurospheres were suspended in a mixture of calpain inhibitor and growth factors (Complete, Roche Diagnostics GmbH, Germany) or PBS/1% glucose medium (i.e., without adding growth factors). ~5 μl of the resulting suspension with a concentration of 2x105 cells/μl was transplanted into the SCI site. Animals received subcutaneous injections of Cyclosporin-A to facilitate survival and were isolated from each other in cages for four weeks. Four weeks after the transplantation of neurospheres, the phenotype, and integration of cells into the SM tissue at the site of injury were analyzed. Growth factors EGF and FGF2 were transplanted into the injured spinal cord in the presence or absence of additional growth factors GF (Complete, Roche Diagnostics GmbH, Germany) (Figure 5 A1-A2, B1-B2).

HUES6- and iPSC-derived neurospheres showed moderate survival when transplanted without growth factors (supplemented with PBS/1% glucose), averaging 80% and 69%, respectively, and significant survival when a cocktail of GF growth factors was applied (on average, 90% and 74%, respectively). Figure 5 shows an example of the survival of HUES6#1-derived neurospheres in the injured spinal cord four weeks after SCI and transplantation. Sections of the microglial scar were immunolabeled with specific human antibodies: hGFAP - human glial fibrillary acidic protein, and hNUC - a nuclear marker associated with neuroblast migration. The morphological picture of the injury site shows that healing occurred better when neurospheres were transplanted with a cocktail of additional GF growth factors. However, it should be noted that the cells derived from HUES6 and iPSCs were primarily located at the edges rather than the epicenter of the injury.

**Figure 6.**
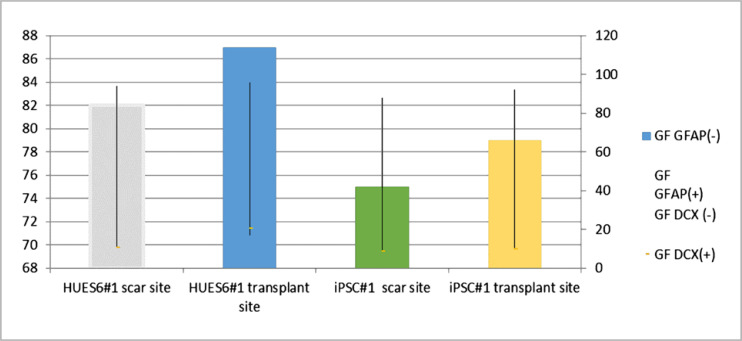
Survival of HUES6#1-derived neurospheres at the fibroglial scar cross-section and microglial after SM injury and transplantation: A - HUES6#1, B - iPSC#1 based on the results of evaluating the expression of the astrocyte marker GFAP and the early neuron marker DCX

To determine the phenotype of transplanted cells derived from PSC in glial and neuronal directions, a quantitative evaluation of the expression of the astrocyte marker GFAP and the early neuronal marker DCX was performed (Figure 6).

The results indicate that despite differences in the cell composition obtained in vitro, neurospheres still retain a certain level of neurogenic potential after in vivo transplantation. Cells that did not fully differentiate into cellular cells in vitro massively approached the GFAP astrocyte phenotype under the influence of the medium into which they were transplanted.

Interestingly, GFAP+ astrocytes in the epicenter or at the edges of the injury also showed the Vimentin marker. In contrast, individual cells that migrated later into the surrounding tissue of the transplant site showed only the astrocyte marker GFAP. Since Vimentin is observed in early-immature astrocytes, it can be assumed that the microenvironment at the same site immediately prevents the maturation of PSC-derived early Vimentin+-astrocytes or induces them to acquire a reactive phenotype.

It should be noted that the neurogenic potential of neurospheres derived from HUES6 and iPSCs was preserved in vivo. However, the injured spinal cord influenced the Vimentin+- and/or GFAP+- astrocytes. Their survival was moderate, mostly limited to the edges of the injury. Neurospheres derived from PSCs from various sources, transplanted into the injured spinal cord of rats, mainly differentiated into astrocytes.

## DISCUSSION

Embryonic stem cells (ESCs) and human iPSCs were chosen as the primary cell base, considering their importance in tissue engineering and regenerative medicine [28-30]. In recent years, there has been a significant advancement in understanding developmental processes, from molecular to in vitro techniques, making most cell types from PSCs achievable [31].

It is known that during the development of the mammalian cerebral cortex, neural stem cells (NSCs) generate neurons and produce glial cells. The mechanism responsible for this developmental shift from neurogenesis to gliogenesis is unknown. Both during in vivo and in vitro expansion, embryonic NSCs can transition from an early neurogenic to a late glycogenic state [32,33]. Unlike astrocytes, which can differentiate from NSCs in several regions of the CNS, the appearance of oligodendrocytes is observed only in some areas of the CNS, for example, in the ventral neural tube [34,35]. It is believed that neurons produced by NSC in vivo program the latter to switch to a glycogenic phenotype using a negative feedback mechanism, which, however, does not occur in the process of NSC expansion in vitro [36-38].

Cheng A. et al. [39] also showed that the increased expression of TRKB-t (tyrosine kinase receptor) in the embryonic cerebral cortex, which co-occurs with the production of astrocytes, plays a vital role in the developmental transition from neurogenesis to gliogenesis. The binding of BDNF to TRKB-t activates a signaling pathway (involving G protein and protein kinase C [40]) that induces NSCs to become glial progenitors and astrocytes.

Sun Y. E. et al. [36] reported that NSCs cultured as monolayers had greater neurogenic potential compared to those cultured as free-floating spheres. Krencik R. et al. proposed a method in which human PSC-derived NSCs are grown as neurospheres in serum-free, low-adhesion conditions for ~160 days [9, 13]. These neurospheres were then differentiated into astrocytes by exposure to CNTF for one week. The authors report that the in vitro glycogenic transition occurred late. They noted that NSCs cultured as huge neurospheres might be more significant than NSCs purified as monolayers due to their three-dimensional structure and complex intercellular interactions. They can maintain their neurogenic potential for more than 200 days in vitro [36].

Worthy of attention are the results obtained in the laboratory of Prof. Winner B. [31], who showed that in vitro maturation of HSCs derived from human PSCs into neurons requires eight weeks. Therefore, only one week of CNTF exposure seems too short for astrocyte maturation in vitro, as confirmed in our study.

Our study showed that differentiation of human PSCs from different sources, according to the protocol published by Krencik R., led to the formation of mixed populations of neurons, astrocytes, and undifferentiated cells in neurospheres at both DIV~80 and DIV~180.

Thus, DIV~180 neurospheres differentiated for two weeks demonstrate a reduced differentiation potential and a higher content of the Sox2 NSC marker than neurospheres. Intense transfer reduces their ability to differentiate in vitro. However, when iPSC-derived and HUES6 neurospheres were transplanted with DIV~130 into the injured rat spinal cord, an average of 74% or 90% of the cells differentiated into GFAP+ astrocytes. In our study, we obtained evidence that neurospheres can give rise to almost pure populations of astrocytes after they have been exposed to the CNS microenvironment in the SCI cell.

The difference in the survival and differentiation of HUES6#1 and iPSC#1 neurospheres, both in vitro and in vivo, can be explained by the different sources of origin of PSCs and the corresponding difference in the behavior of signaling molecules in umbilical cord blood PSCs and iPSCs under the influence of epigenetic memory in the process of reprogramming differentiated cells on induced PSCs.

We agree with Vierbuchen T. and Wernig M. [41], who believe that the degree of influence of epigenetic memory on the functional properties of reprogrammed cells is the molecular basis. However, the impact of reprogramming techniques on epigenetic memory requires further research.

Small Rho GTPases may be signaling molecules associated with epigenetic memory. They activate the ROCK kinase and control neural cell migration, proliferation, death, and reactive astrogliosis [42, 43].

The Rho/ROCK signaling pathway is known to mediate the effects of endogenous inhibitors of axonal growth from myelin, oligodendrocytes, and glial scar tissue. It is activated upon spinal cord injury, leading to increased inflammatory response, development of neuropathic pain, demyelination, cell death, inhibition of axonal growth, and impaired function [38]. After SM damage, the extracellular space becomes an obstacle to the regeneration of neurons, including forming a growth cone at the proximal end of the damaged axon. However, the structures of the glial scar express both inhibitors of axon growth and molecules that have the opposite effect: they stimulate the growth and regeneration of axons [44]. Therefore, intracellular modulation of exogenous Rho proteins, which control the movement of cells and other regulatory molecules, despite extracellular signals that promote apoptosis and growth cone collapse, may explain the regeneration of CM at the site of injury.

## CONCLUSION

The findings of our study, which followed the established protocol for differentiating human PSC derivatives, did not reveal the maturation of NSCs into astrocyte populations and did not confirm the existence of a glycogenic transition of PSC-derived NSCs in vitro.

However, a significant influence of the environment of the injured spinal cord on PSC differentiation after transplantation was proven, and it was shown that HUES6-derived neurospheres gave rise to 90% of GFAP+ astrocytes and 5–10% of DCX+ early neurons in vivo. In all animals transplanted with iPSC#1 neurospheres, 74% of the transplanted cells differentiated into GFAP+ astrocytes, while 5% of the cells showed the early neuronal marker DCX+.

A possible explanation for the difference in survival and differentiation of HUES6#1 and iPSC#1 neurospheres both in vitro and in vivo may be a difference in the regulatory behavior of signaling molecules associated with epigenetic memory, depending on the source of origin of PSCs.

The results showed that the transplantation of neurospheres into the damaged spinal cord could be a source of cellular elements necessary for the activation of neurogenesis, the formation, and the proliferation of various types of nerve cells, mainly astrocytes. It is promising to use neurospheres as a tool for studying experimental models of nervous system pathology.
